# MiR-1-3p targets CENPF to repress tumor-relevant functions of gastric cancer cells

**DOI:** 10.1186/s12876-022-02203-2

**Published:** 2022-03-28

**Authors:** Shenkang Zhou, Hui Han, Leilei Yang, Hui Lin

**Affiliations:** 1 Department of Gastrointestinal Surgery, Taizhou Hospital, Zhejiang University, Taizhou City, Zhejiang Province People’s Republic of China; 2 Key Laboratory of Minimally Invasive Techniques and Rapid Rehabilitation of Digestive System Tumor of Zhejiang Province, Taizhou City, People’s Republic of China; 3 School of Medicine, Zhejiang University, Hangzhou City, Zhejiang Province People’s Republic of China; 4 Department of General Surgery, The Second Affiliated Hospital of Shantou Medical College, Shantou City, Guangdong Province People’s Republic of China; 5 Department of General Surgery, Sir Run Run Shaw Hospital, School of Medicine, Zhejiang University, No. 3 Qingchun East Road, Hangzhou City, 310016 Zhejiang Province People’s Republic of China

**Keywords:** Gastric cancer, miR-1-3p, CENPF, Proliferation, Invasion and migration

## Abstract

Here we noted significantly downregulated miR-1-3p in gastric cancer (GC) tissue compared with adjacent normal tissue through qRT-PCR. Lowly expressed miR-1-3p correlated GC progression. Overexpressing miR-1-3p could restrain tumor-relevant cell behaviors in GC, while miR-1-3p inhibitor treatment triggered the opposite results. Moreover, dual-luciferase reporter gene detection identified specific binding sites of miR-1-3p in CENPF 3’untranslated region. Upregulating miR-1-3p constrained cell progression of GC via CENPF downregulation. Western blot, qRT-PCR and dual-luciferase detections manifested that miR-1-3p negatively mediated CENPF expression in GC cells. Thus, we demonstrated that miR-1-3p negatively mediated CENPF to hamper GC progression. CENPF may be an underlying target for GC therapy.

## Introduction

Gastric cancer (GC) tumor originates from gastric mucosal epithelium. Patient’s age is approximately 50 years old. The incidence of GC exhibits a younger trend due to the changed diet, increased working pressure and H pylori infection. GC, mostly adenocarcinoma, may occur everywhere in the stomach [1]. In clinical, the lack of special symptoms are pain points for patient’s early diagnosis. As the diagnostic and therapeutic levels for patients in the early-stage increase, their long-term survival rate is enhanced but the prognosis is still dismal for those in the advanced stage [2]. Therefore, further elaboration of GC tumor onset and metastasis remains to be done.

Frequent aberrant gene expression quickens occurrence of human malignant tumors. Centromere Protein F (CENPF) is a nuclear antigen pertinent to cell cycle. CENPF correlates filament-mitochondrial complex, presenting impacts on cancer progression [3]. Highly expressed CENPF was observed in various human malignant tumors, such as GC [4], prostate cancer [5], breast cancer [3] and hepatocellular cancer [6]. Besides, its stimulatory impacts have been elucidated in tumors. The typical examples are the positive relevance of CENPF overexpression to malignancy and bad prognosis of prostate cancer [5], as well as bone metastasis in breast cancer [3]. These remind us of the promotion of CENPF in tumor onset and progression. Nevertheless, how CENPF works in GC is far less understood.

MiR-1-3p is one of the mature RNA molecules and has been extensively investigated in cancer research. It was considered as a tumor-suppressing gene in hepatocellular cancer [7], renal cell carcinoma [8] and bladder carcinoma [9]. For instance, miR-1-3p attenuated the proliferation ability of hepatocellular carcinoma by inhibiting SOX9 expression [7]. Also, the roles of miR-1 in GC have been studied to some degree. Recently, in some studies, it was concluded that miR-1 suppresses GC cell migration ability as well as prevents multidrug resistance happening in GC cells [10, 11]. Although several GC studies have focused on miR-1, more comprehensive clarifies are needed in the underlying mechanisms of miR-1-3p in GC.

Here, combing bioinformatics analyses, a set of molecular and cellular experiments were introduced to comprehensively verify the role of miR-1-3p in GC, and the effects of miR-1-3p/CENPF axis on GC was firstly determined.

## Materials and methods

### Bioinformatics analysis

Mature miRNA data (normal: 45, tumor: 446) and Counts data of mRNAs (normal: 32, tumor: 375) in The Cancer Genome Atlas-Stomach Adenocarcinoma (TCGA-STAD) were gained from TCGA. Then, CENPF expression was examined by *t*-test in normal tissue and cancer tissue. Differential expression analysis was carried out on mRNAs with “EdgeR” package (|logFC|> 2.0, padj < 0.01). starBase (http://starbase.sysu.edu.cn/) and miRTarBase (http://mirtarbase.mbc.nctu.edu.tw/php/index.php) databases were utilized to predict downstream regulatory mRNAs of miR-1-3p. An intersection was taken between the predicted miRNAs and differentially downregulated miRNAs. Pearson correlation analysis was undertaken on miRNAs and CENPF. MiRNA expression was further explicated by *t*-test.

### Cell culture and cell transfection

Normal human gastric epithelial cell line GES1 (BNCC337969), GC cell lines AGS (BNCC338141), SGC-7901 (BNCC100114) and MGC803 (BNCC100665) were procured from BeNa Culture Collection (BNCC). GES1 and MGC803 were grown in Dulbecco’s Modified Eagle Medium (DMEM) + 10% fetal bovine serum (FBS). AGS cell line was cultivated in F-12 complete medium + 10% FBS. SGC-7901 cell line was grown in Roswell Park Memorial Institute (RPMI) complete medium. They were all preserved in standard cultural condition. RiboBio provided miR-1-3p inhibitor, miR-1-3p mimic (miR-mimic), pcDNA3.1-CENPF plasmids (oe-CENPF) encoding CENPF and corresponding negative controls (NCs). These plasmids were transfected to designated cell lines with lipofectamine RNAiMAX (Life Technologies).

### qRT-PCR

Total RNA was extracted from cells using Trizol kit. MiRNA (10 μg) and mRNA were reversely transcribed into cDNA by Taq-Man®MicroRNA (TAKARA) and One step RT-PCR Kit (OMEGA), respectively. qRT-PCR was detected by SYBR Green on CFX Connet TM Real-time PCR system. GAPDH or U6 mRNA was taken for standardization. Primer sequences were as follows: miR-1-3p, forward: 5′-CAGTGCGTGTCGTGG AGT-3′, reverse: 5′-GGCCTGGAATGTAAAGAAGT -3′; U6, forward: 5′-CTCGCTTCGGCAGCACA-3′, reverse: 5′-AACGCTTCACGAATTTGCGT-3′; CENPF forward: 5′- AAAGAAACAGACGGAACAACTG -3′, reverse: 5′-CCAAGCAAAGACCGAGAACT -3′; GAPDH forward, 5′-ACATCGCTCAGACACCATG-3′, reverse, 5′-TGTAGTTGAGGTCAATGAAGGG-3′. MiRNA and mRNA fold changes were calculated by 2^−ΔΔCt^.

### Western blot assay

Extraction of Total proteins was conducted using radio immunoprecipitation assay buffer and equivalent proteins were separated on 10% sodium dodecyl sulfate polyacrylamide gel electrophoresis. Then, proteins were moved to polyvinylidene fluoride membrane. After being blocked at room temperature for an h, the membrane was first incubated with primary antibodies rabbit anti-CENPF (1:1500, ab5, Abcam, China) and rabbit anti-GAPDH (1:1000, ab8245, Abcam, China), and then secondary antibody goat anti-rabbit IgG H&L (HRP) (ab6721, Abcam, China). Afterwards, the membrane was rinsed three times with Tris buffered saline/Tween-20 (TBST) and tested on chemiluminescence system.

### Cell proliferation and colony formation experiments

Cells transfected with miR-1-3p inhibitor, miR-1-3p mimic, oe-CENPF and corresponding NC were inoculated in 96-well plates at 3000 cells/well. Cell proliferative ability was measured at 0, 24, 48, 72 and 96 h. The medium was abandoned at a specific time and cell counting kit 8 (CCK-8) was instilled to each well for 2 h incubation following the manufacturer’s procedure. Cell proliferation was detected at 450 nm wavelength.

Cells were resuspended in RPMI-1640 + 10% FBS and seeded in 12-well plates at 300 cells/well. 14 d later, cells were fixed with methyl alcohol for 15 min and stained utilizing crystal violet for 20 min. Cell colonies were counted. The procedure was subjected to 3 repetitions.

### Cell migration assay

In cell migration assay, MGC803 and AGS cells were first planted in 12-well plates. As cell coverage reaching 80%, the central single layer was scraped by a 200 μL pipette tip. Wells were briefly washed twice by medium to get rid of separate cells. Fresh medium was added for another 24 h of cell growth. Cell migration was viewed and photographed at 0 h and 24 h on microscopy. This procedure was repeated in triplicate.

### Cell invasion assay

Transwell invasion determination was undertaken on a 24-well Transwell chamber coated with Matrigel in advance. Cells were transfected with plasmids described above. Twenty-four h later, 300 µl cell suspension was supplemented to the upper chamber and 500 µl medium with 10% FBS was added to the lower chamber. Thereafter, cells were incubated for one day. Cells in the upper chamber were abandoned, while those in the lower chamber were fixed and dyed by crystal violet for 24 h. In at least 5 random fields, cells were determined through a microscopy.

### Dual-luciferase assay

psiCHGCK luciferase reporter plasmids (Sangon Co., LTD, Shanghai, China) inserted with CENPF mutant (mut) and wild type (wt) 3’-untranslated region (UTR) were constructed to identify the binding between two studied genes. Subsequently, GC cells were inoculated in 48-well plates for 24 h of culture at 37 ℃. MiR-1-3p mimic/mimic NC and CENPF-psiCHGCK wt/mut plasmids were co-transfected to GC cells. Finally, luciferase assay reagent (Promega, Fitchburg, WI, USA) was utilized to examine luciferase intensity.

### Statistical analysis

This step was taken on SPSS 19.0, and outcomes were visualized on Prism 6.0. All information was displayed as mean ± standard deviation (SD). Aaasys were independently performed 3 times at least. Student’s *t* test was conducted to determine statistical significance. *P* value smaller than 0.05 denoted a significance.

## Results

### MiR-1-3p expression is low in GC

**Fig. 1:**
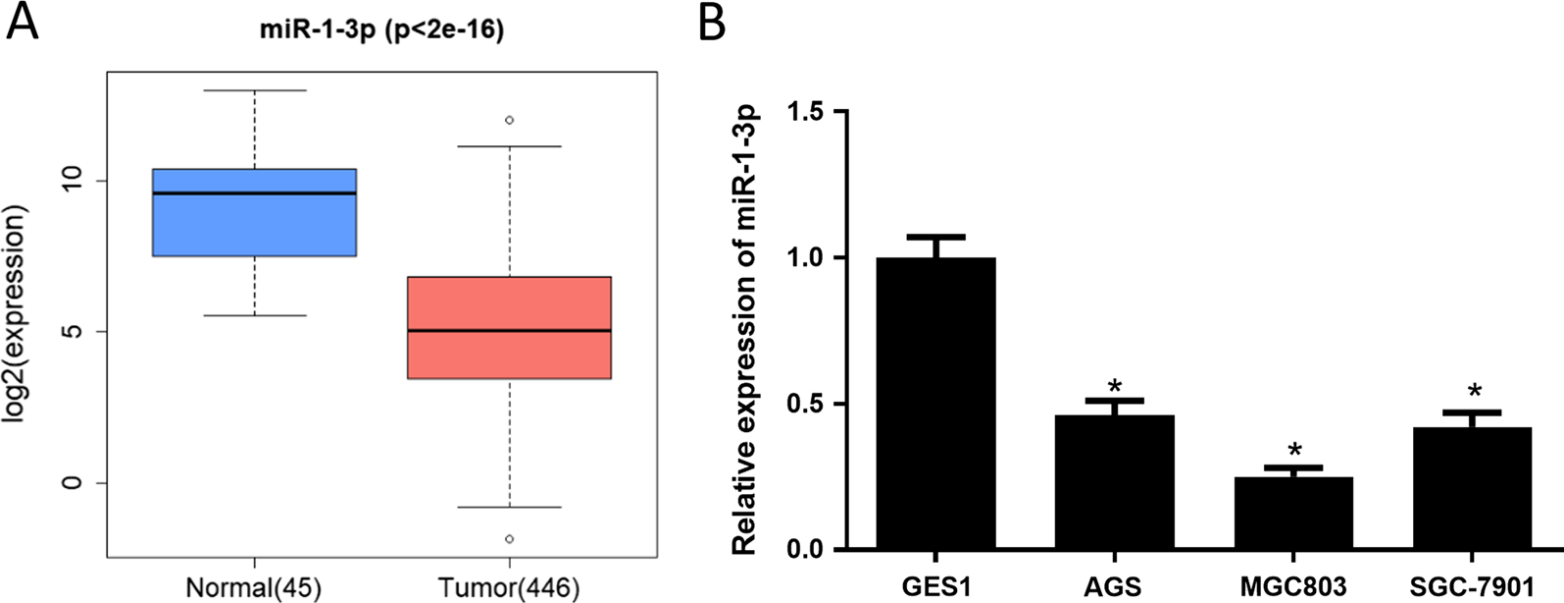
MiR-1-3p is downregulated in GC tissue and cells. **A** Compared with normal tissue, miR-1-3p was markedly down-regulated in tumor tissue according to data from TCGA and analyzed by two-tailed test; **B** MiR-1-3p level at cellular level. *p < 0.05

miRNA expression profiles of normal and cancer tissue were accessed from TCGA. MiR-1-3p level in GC tissue was significantly low (Fig. 1A). Aberrant miR-1-3p expression was demonstrated to influence varying cancers [7–9, 12], thus it was chosen in the present study. MiR-1-3p expression was remarkably downregulated in GC cell lines, especially in MGC803 and AGS cell lines (Fig. 1B). Thus, MGC803 and AGS were chosen for the subsequent functional analyses.

### MiR-1-3p overexpression hampers GC cell functions

**Fig. 2:**
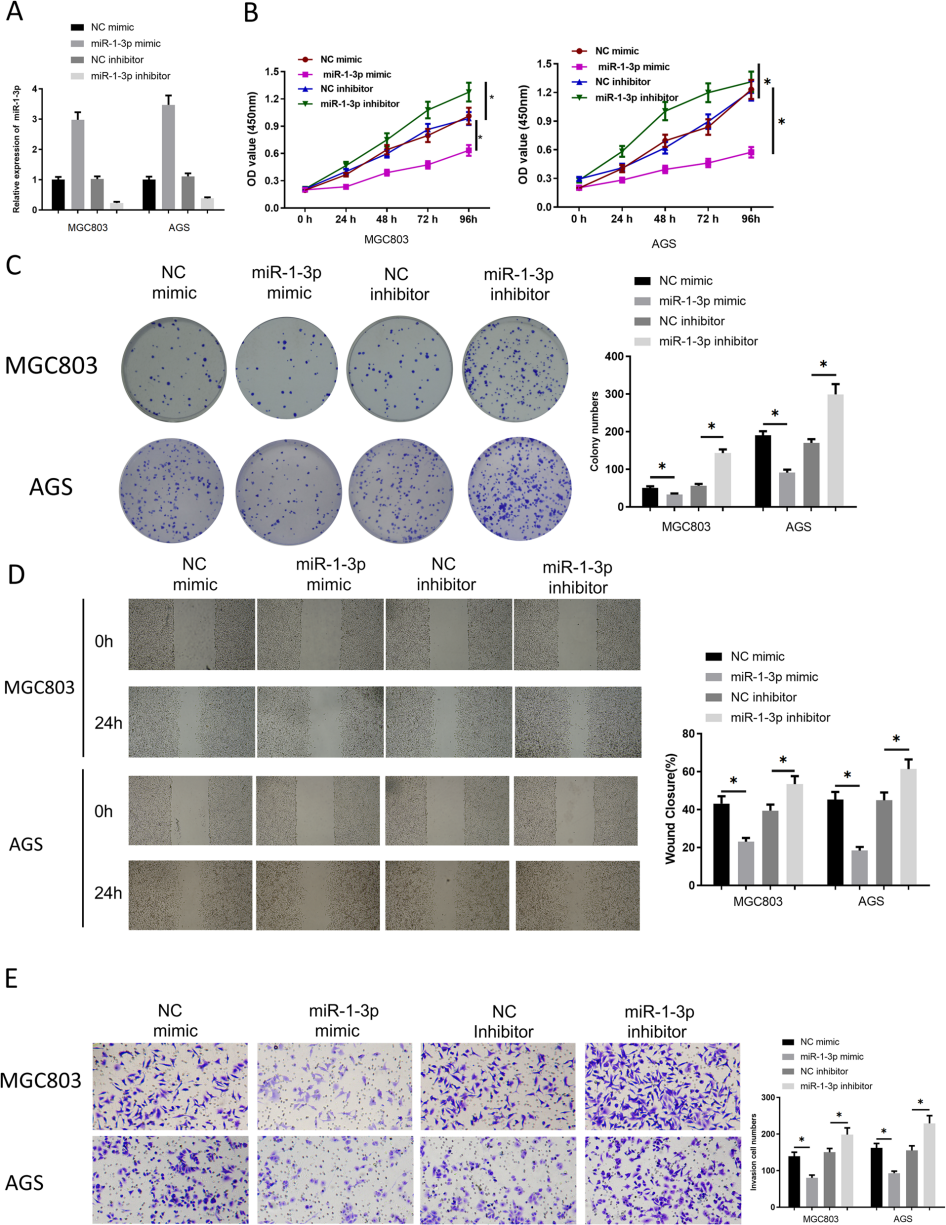
MiR-1-3p overexpression hampers GC cell proliferation, invasion and migration. **A** Level of miR-1-3p upon transfection; **B**–**E** Cell functions after transfection were measured by CCK-8, colony formation, wounding healing (40 ×) and Transwell (100 ×) methods. *p < 0.05

MiR-1-3p was overexpressed/inhibited in MGC803 and AGS cells to analyze its impact on cell proliferation, invasion and migration. qRT-PCR discovered that miR-1-3p mimic transfection elevated miR-1-3p level in GC cells while miR-1-3p inhibitor transfection triggered the opposite results (Fig. 2A). According to CCK-8, colony formation, wound healing and Transwell methods, overexpressing miR-1-3p declined cell proliferative rate, migration and invasion, while suppressing this gene led to the opposite results (Fig. 2B–E). On the above, miR-1-3p hampered GC cell malignant behaviors in vitro.

### CENPF is a direct target of miR-1-3p

**Fig. 3:**
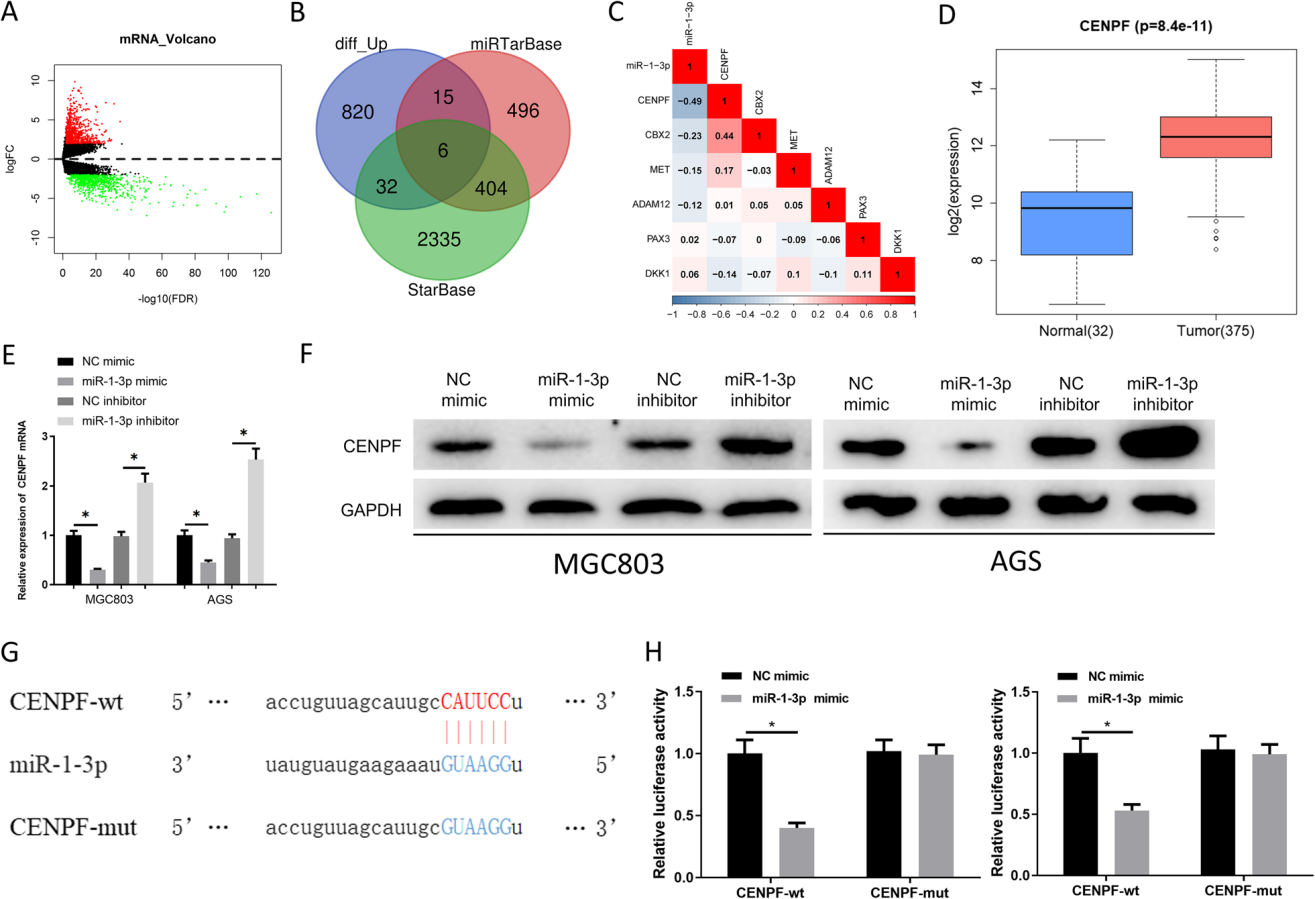
CENPF is a direct target of miR-1-3p. **A** Volcano plot of DEmRNAs in TCGA-STAD. Red: differentially upregulated genes; green: differentially downregulated genes; **B** Venn diagram of predict upregulate genes and target genes; **C** Pearson correlation analysis between miR-1-3p and 6 target mRNAs; **D** Upregulated CENPF as tested by two-tail test; **E**, **F** mRNA and protein levels of CENPF in each group; **G** Putative binding sites of CENPF 3’UTR and miR-1-3p. Mutations were generated in the 3′-UTR of CENPF by mutated seed matching sequences; **H** Dual-luciferase detected luciferase activity in co-transfected cells. *p < 0.05

A total of 1642 differentially expressed mRNAs (DEmRNA) (upregulated: 873, downregulated: 769) were obtained from TCGA (Fig. 3A). The targets of miR-1-3p were bioinformatically predicted by miRTarBase and StarBase databases, followed by overlapping the up-regulated DEmRNAs with the predicted mRNAs. Consequently, 6 mRNAs were acquired (Fig. 3B). Afterwards, the association between these 6 target genes and miR-1-3p was analyzed. CENPF presented a remarkably negative association with miR-1-3p (Fig. 3C) and conspicuous upregulation in GC tissue and cells (Fig. 3D). MiR-1-3p overexpression hampers CENPF level while its inhibition increased CENPF level (Fig. 3E, F). Publicly available algorithm TargetScan predicted target of miR-1-3p (Fig. 3G). The 3’-UTR of CENPF mRNA fragment was established using target sequences and then cloned into psiCHECK-2 luciferase reporter carriers as well as mutant CENPF mRNA 3′-UTR. It turned out that miR-1-3p mimic markedly declined the luciferase intensity of cells with psiCHECK-2 3′-UTR-CENPF-wt while did not decline that of cells with psiCHECK-2-3′-UTR-CENPF-mut (Fig. 3H). On the whole, CENPF was regulated by miR-1-3p.

### MiR-1-3p modulates GC cell progression via targeting CENPF

**Fig. 4:**
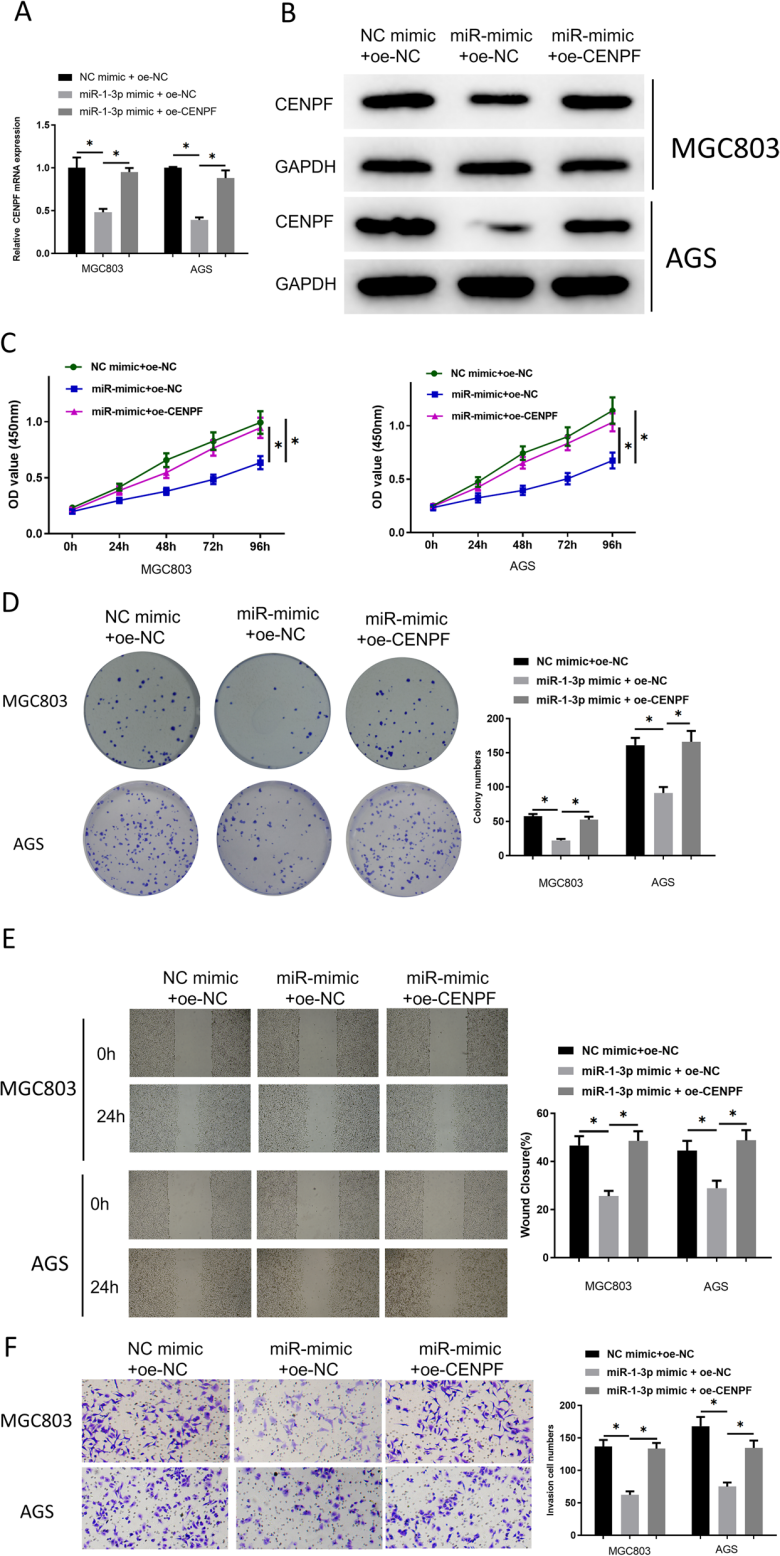
MiR-1-3p modulates GC cell functions through targeting CENPF. **A**, **B** qRT-PCR and western blot were used to measure the mRNA and protein expression levels of CENPF in MGC803 and AGS cells in each group; **C**–**F** GC cell functions after transfection were measured by CCK-8, colony formation, wounding healing (40 ×) and Transwell (100 ×) methods. *p < 0.05

Transfection groups were built to further understand whether CENPF mediates the impact of miR-1-3p on cell functions (Fig. 4A, B). CCK-8 analysis revealed the inhibition of miR-1-3p aberrant expression on cell proliferation. CENPF upregulation in MGC803 and AGS could conspicuously reverse this impact (Fig. 4C). Colony formation analysis manifested that miR-1-3p overexpression in MGC803 and AGS cells decreased cell colonies, but this impact could be counteracted by CENPF upregulation (Fig. 4D). In addition, miR-1-3p mimic transfection could weaken cell migratory and invasion abilities, while increasing CENPF could attenuate the impact (Fig. 4E, F). Therefore, these results suggested that upregulated CENPF partly reversed the impacts of miR-1-3p on GC cell functions.

## Discussion

Despite benefits of chemotherapy and targeted therapy (trastuzumab), tumor metastasis remains major pain point [13]. To date, the mechanism of GC is elusive and needs to be researched. Here, overexpressed miR-1-3p was revealed to be a suppressor.

MiRNAs usually facilitate or suppress cancer cell progression by mediating downstream target genes [14]. Frequently dysregulated miRNAs in human cancers hint at their functions as key regulators [15]. Thus, it was raised that miRNAs may be effective targets for GC. Many studies verified that miR-1 and miR-1-3p were downregulated in many malignant tumors and their overexpression suppressed tumor progression in GC [1, 10, 11, 16, 17]. Likewise, this work also determined tumor-suppressing roles of miR-1-3p in GC and evaluated its downstream mechanisms. Ke and his colleagues proposed the tumor-promoting mechanism of miR-1-3p/STC2 axis in GC [1]. In another study, Peng Deng determined LINC00242/miR-1-3p/G6PD regulatory axis in aerobic glycolysis in GC [17]. Different from the studies mentioned above, we screened the CENPF, a target of miR-1-3p, and identified the tumor-suppressing effects of miR-1-3p/CENPF axis. To sum up, though miR-1-3p has been widely understood as a tumor-suppressor [18], the downstream mechanisms of miR-1-3p have not been fully revealed, and our study provided one of the mechanisms on miR-1-3p in GC.

CENPF is a crucial centromere—mitochondrial complex protein in cell division of somatic cells [19]. This microtubule-binding protein mediates pyruvate kinase M2 phosphorylation signaling via tumor metabolism regulation [5]. CENPF facilitates tumor proliferation and metastasis. For instance, dysregulation of miRNAs-COUP-TFII-FOXM1-CENPF axis is conductive to metastasis of prostate cancer [20]. Downregulated CENPF remodels prostate cancer and changes cell metabolism [21]. Thus, CENPF serves as a putative target of cancer treatment. Moreover, miRNAs can inhibit tumor growth via targeting CENPF. For example, miR-28-5p attenuates breast cancer progression via targeting CENPF [22]. Haiting Xu pointed out suppressing effects of miR-383-5/CENPF axis in breast cancer [23]. Nonetheless, the interplay of miR-1-3p and CENPF remains elusive in GC cells. Here, CENPF was discovered to be negatively correlated with miR-1-3p. Overexpressing miR-1-3p remarkably hampered CENPF mRNA and protein levels in MGC803 and AGS. Rescue assay illuminated that CENPF overexpression recovered the inhibition of miR-1-3p. On the above, miR-1-3p targeted CENPF to hamper GC cell functions possibly by influencing cell division.

On the whole, miR-1-3p mediated GC development via CENPF modulation. Lowly expressed miR-1-3p correlated GC tumor proliferation, invasion and migration. The two may be crucial targets in GC therapy. However, the cause inducing downregulation of miR-1-3p in GC has not been identified in our study yet, which may be one of the limitations. In the study conducted by Peng Deng, LINC00242 was considered as a suppressor for miR-1-3p in GC, indicating that overexpression of LINC00242 could promote malignancy of GC [17]. Based on Deng’s study, we are assuming LINC00242/miR-1-3p/CENPF axis could also regulate GC cells in a similar way.

## Data Availability

The data and materials in the current study are available from the corresponding author on reasonable request.

## References

[1] Ke J (2019). MiR-1-3p suppresses cell proliferation and invasion and targets STC2 in gastric cancer. Eur Rev Med Pharmacol Sci.

[2] Xu X, Zhang Y, Liu Z, Zhang X, Jia J (2016). miRNA-532-5p functions as an oncogenic microRNA in human gastric cancer by directly targeting RUNX3. J Cell Mol Med.

[3] Sun J (2019). Overexpression of CENPF correlates with poor prognosis and tumor bone metastasis in breast cancer. Cancer Cell Int.

[4] Chen EB (2019). HnRNPR-CCNB1/CENPF axis contributes to gastric cancer proliferation and metastasis. Aging (Albany NY).

[5] Shahid M (2018). Centromere protein F (CENPF), a microtubule binding protein, modulates cancer metabolism by regulating pyruvate kinase M2 phosphorylation signaling. Cell Cycle (Georgetown, Tex.).

[6] Kim HE (2012). Frequent amplification of CENPF, GMNN and CDK13 genes in hepatocellular carcinomas. PLoS ONE.

[7] Zhang H (2019). miR-1-3p suppresses proliferation of hepatocellular carcinoma through targeting SOX9. Onco Targets Ther.

[8] Liu J (2019). miR-1-3p suppresses the epithelial-mesenchymal transition property in renal cell cancer by downregulating Fibronectin 1. Cancer Manag Res.

[9] Gao L, Yan P, Guo FF, Liu HJ, Zhao ZF (2018). MiR-1-3p inhibits cell proliferation and invasion by regulating BDNF-TrkB signaling pathway in bladder cancer. Neoplasma.

[10] Lin XQ (2020). mir-1 inhibits migration of gastric cancer cells. Front Biosci (Landmark edition).

[11] Deng LM, Tan T, Zhang TY, Xiao XF, Gu H (2019). miR-1 reverses multidrug resistance in gastric cancer cells via downregulation of sorcin through promoting the accumulation of intracellular drugs and apoptosis of cells. Int J Oncol.

[12] Li SM (2018). The putative tumour suppressor miR-1-3p modulates prostate cancer cell aggressiveness by repressing E2F5 and PFTK1. J Exp Clin Cancer Res.

[13] Wang R (2019). Downregulation of miRNA-214 in cancer-associated fibroblasts contributes to migration and invasion of gastric cancer cells through targeting FGF9 and inducing EMT. J Exp Clin Cancer Res.

[14] Luo J, Pan J, Jin Y, Li M, Chen M (2019). MiR-195-5p inhibits proliferation and induces apoptosis of non-small cell lung Cancer cells by targeting CEP55. Oncol Targets Ther.

[15] Luo Q (2014). MicroRNA-195-5p is a potential diagnostic and therapeutic target for breast cancer. Oncol Rep.

[16] Han C (2015). MicroRNA-1 (miR-1) inhibits gastric cancer cell proliferation and migration by targeting MET. Tumour Biol.

[17] Deng P (2021). LINC00242/miR-1-3p/G6PD axis regulates Warburg effect and affects gastric cancer proliferation and apoptosis. Mol Med (Cambridge, Mass.).

[18] Han C, Shen JK, Hornicek FJ, Kan Q, Duan Z (2017). Regulation of microRNA-1 (miR-1) expression in human cancer. Biochim Biophys Acta Gene Regul Mech.

[19] Toralova T, Susor A, Nemcova L, Kepkova K, Kanka J (2009). Silencing CENPF in bovine preimplantation embryo induces arrest at 8-cell stage. Reproduction.

[20] Lin SC (2016). Dysregulation of miRNAs-COUP-TFII-FOXM1-CENPF axis contributes to the metastasis of prostate cancer. Nat Commun.

[21] Shahid M (2019). Downregulation of CENPF remodels prostate cancer cells and alters cellular metabolism. Proteomics.

[22] Chen Q, Xu H, Zhu J, Feng K, Hu C (2020). LncRNA MCM3AP-AS1 promotes breast cancer progression via modulating miR-28-5p/CENPF axis. Biomed Pharmacother.

[23] Xu H, Zhu X, Shi L, Lin N, Li X (2021). miR-383-5p inhibits human malignant melanoma cells function via targeting CENPF. Reprod Biol.

